# Macrophage Activation Syndrome (MAS): A Case Report and Narrative Review

**DOI:** 10.7759/cureus.35670

**Published:** 2023-03-01

**Authors:** Arthur Dilibe, Onyinye S Ugoala, Endurance O Evbayekha, Mohammad Z Khalilullah, Olanrewaju K Adabale, Tracy-Ann Poyser, Osejie F Oriaifo, Ufuoma I Olori, Henry O Aiwuyo

**Affiliations:** 1 Internal Medicine, Brody School of Medicine at East Carolina University, Greenville, USA; 2 Internal Medicine, College of Medicine, University of Lagos, Lagos, NGA; 3 Internal Medicine, St. Luke's Hospital, Chesterfield, USA; 4 Internal Medicine, Unity Health White County Medical Center, Searcy, USA; 5 Internal Medicine, Jersey City Medical Center, Jersey City, USA; 6 Internal Medicine, Brookdale University Hospital Medical Center, Brooklyn, USA

**Keywords:** macrophage activation syndrome, adult-onset still’s disease, hemophagocytic lymphohistiocytosis, uti, sepsis, adult-onset still’s disease (aosd), macrophage activation syndrome (mas), hemophagocytic lymphohistiocytosis (hlh)

## Abstract

Hemophagocytic lymphohistiocytosis (HLH) is a rare and life-threatening syndrome of excessive inflammation and tissue destruction secondary to abnormal immune activation. The term macrophage activation syndrome (MAS) is used when HLH develops in the setting of systemic juvenile idiopathic arthritis (SJIA; formerly known as Still’s disease), adult-onset Still’s disease, or any other rheumatologic disorder. We present a case of a 21-year-old female with a known history of SJIA who presented to the hospital with fever, chills, myalgia, nausea, vomiting, and hypotension. Initial evaluation at the time of presentation suggested sepsis likely due to acute pyelonephritis, and the patient was started on antibiotics and intravenous fluid hydration. However, further workup suggested that her symptoms were non-infectious and were likely due to MAS, a rare complication of SJIA. We promptly diagnosed her, and she received a course of steroids and made an uneventful recovery.

## Introduction

Hemophagocytic lymphohistiocytosis (HLH) is a rare and life-threatening syndrome of excessive inflammation and tissue destruction secondary to abnormal immune activation [1]. Macrophage activation syndrome (MAS) is used when HLH develops in the setting of systemic juvenile idiopathic arthritis (SJIA; formerly known as Still’s disease), adult-onset Still’s disease (AOSD), or any other rheumatologic disorder. MAS typically develops within the first week of the onset of the rheumatologic disorder, but it can occur at any point in the course of the disease [1]. MAS can be difficult to diagnose and requires a high index of suspicion. Fever, elevated ferritin, elevated acute inflammatory markers, cytopenias, and hepatosplenomegaly in the setting of associated underlying disease generally characterize the syndrome. We report a case of MAS as a rare complication of SJIA [1].

## Case presentation

A 21-year-old female with a pertinent medical history of systemic juvenile rheumatoid arthritis, dermatomyositis, developmental delay, and class 3 obesity presented to the emergency department (ED) via emergency medical services (EMS) with fever, chills, myalgia, nausea, vomiting, and hypotension (during transit by EMS). On arrival at the ED, her heart rate was markedly elevated to 164 beats per minute. Her respiratory rate was 23 breaths per minute with an oxygen saturation of 98% on ambient air. She was observed to have a high-grade fever with a temperature of 103.1°F. However, she became normotensive in the ED with a blood pressure of 117/75 mmHg.

On examination, she was confused, ill-appearing, and in significant distress. On the respiratory exam, she was clear to auscultate bilaterally, and her cardiovascular exam was only pertinent for tachycardia. Her abdomen was distended and mildly tender throughout, without rebound or guarding. On neurological exam, no focal deficits were observed, and her review of systems was negative for headaches, cough, nausea, vomiting, chest pain, abdominal pain, diarrhea, or constipation.

The initial labs demonstrated leukocytosis with neutrophilic predominance, microcytic anemia, and smudge cells on a peripheral blood smear (Table 1).

**Table 1:** Initial complete blood count (CBC) results at presentation

Parameter	Results	Reference range
White blood cell count	18.21	4.50 - 11.00 k/uL
Red blood cell count	3.69	3.80 - 5.20 M/uL
Hemoglobin	8.8	12.0 - 16.0 g/dL
Mean corpuscular volume	78.9	80.0 - 100.0 fL
Platelet count	305	150 - 440 k/uL
Absolute neutrophil count	12.56	1.80 - 7.70 k/uL
Peripheral blood smear	Few smudge cells present	None

Her basic chemistry result was pertinent for hypotonic hypovolemic hyponatremia, anion gap metabolic acidosis with elevated lactic acid, elevated inflammatory markers, and transaminitis with a hepatocellular pattern (Table 2). Urinalysis showed pyuria and bacteria but was negative for leukocyte esterase and nitrites (Table 3).

**Table 2:** Basic chemistry results at presentation

Parameter	Results	Reference range
Sodium	128	136 - 145 mEq/L
Potassium	3.4	3.4 - 4.4 mEq/L
Chloride	100	98 - 107 mEq/L
Bicarbonate	14	22 - 29 mEq/L
Anion gap	14	8 - 12 mEq/L
Alkaline phosphatase	39	40 - 150 U/L
Aspartate aminotransferase	194	5 - 34 U/L
Alanine aminotransferase	72	0 - 55 U/L
Lactic acid	3.5	0.5 - 2.0 mmol/L
Creatine kinase	1323	29 - 168 U/L
High-sensitivity C-reactive protein	40.7	<5 mg/L
Erythrocyte sedimentation rate	83	<20 mm/h
Triglyceride	229	<=150

**Table 3:** Urinalysis results at presentation HPF: high power field.

Component	Results	Reference range
Appearance, urine	Cloudy	Clear
Color, urine	Straw	Colorless, yellow, straw
pH, urine	6.5	5.0 - 8.0
Specific gravity, urine	1.005	1.000 - 1.050
Protein, urine	Trace	Negative
Hemoglobin, urine	3+	Negative
Leukocyte esterase, urine	Negative	Negative
Nitrites, urine	Negative	Negative
RBC, urine	1	0-5/HPF
WBC, urine	4	0-3/HPF
Squamous epithelial cells, urine	Few	None seen/HPF
Bacteria, urine	Present	None seen

**Figure 1:**
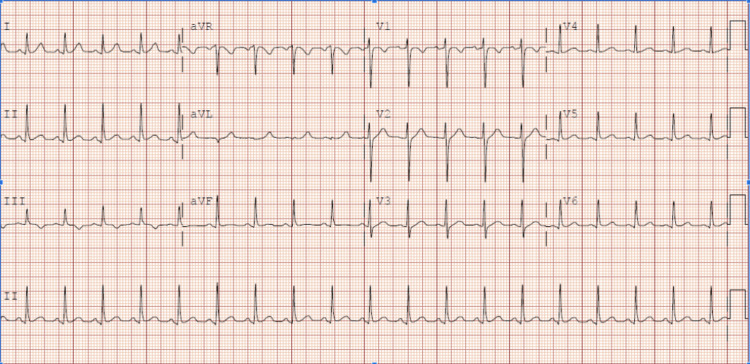
Electrocardiograph (ECG) at presentation showing sinus tachycardia

Her respiratory polymerase chain reaction (PCR) panel (including coronavirus disease 2019) and hepatitis viral panel were all negative. Her chest X-ray was unremarkable, and her electrocardiograph (ECG) (Figure 1) showed sinus tachycardia with a heart rate of 148 beats per minute.

**Figure 2:**
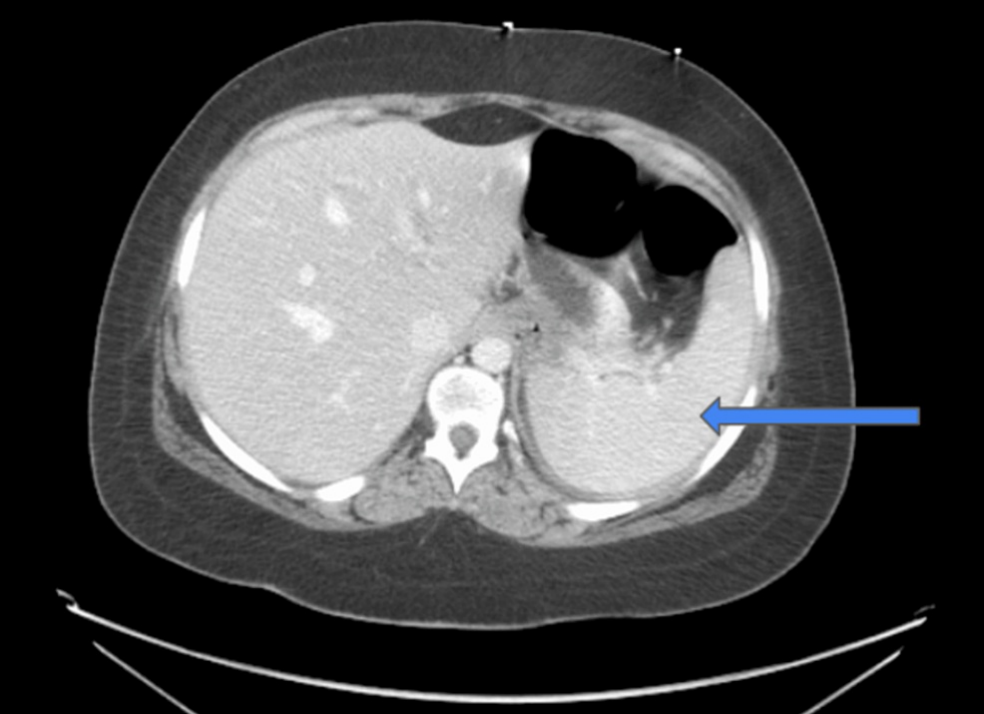
CT of the abdomen/pelvis with IV contrast at presentation showed mild splenomegaly (blue arrow)

**Figure 3:**
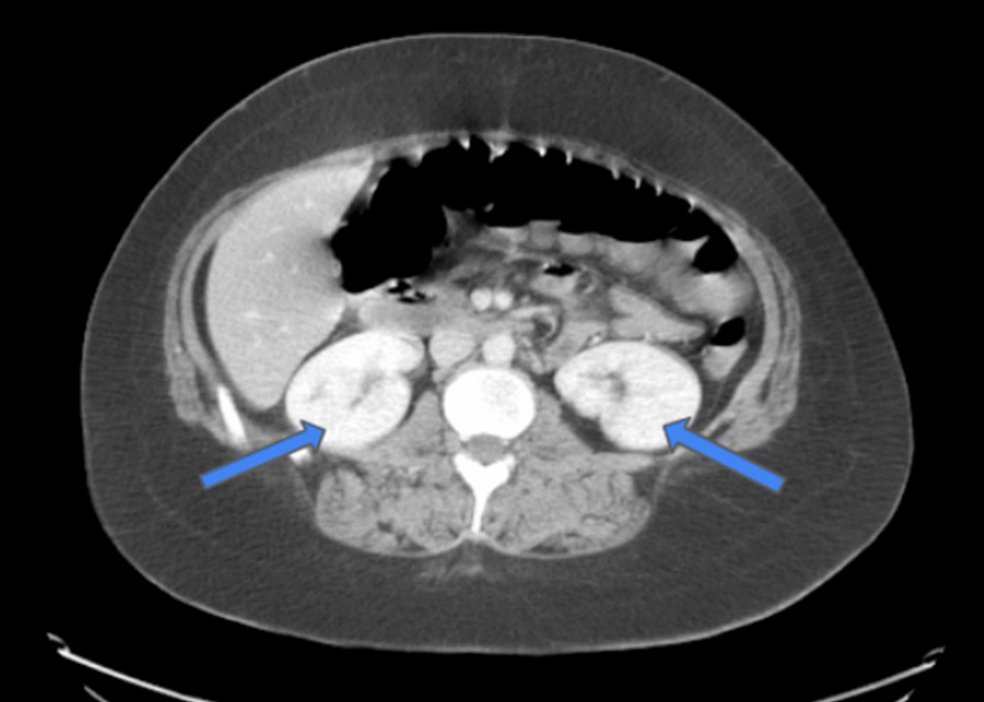
CT of the abdomen/pelvis with IV contrast at presentation showed bilateral multifocal scarring of the kidneys with superimposed pyelonephritis (blue arrows)

CT of the abdomen and pelvis with IV contrast revealed mild splenomegaly (Figure 2) and bilateral multifocal scarring of the kidneys with superimposed pyelonephritis (Figure 3).

Of note, four days before this hospital admission, the patient was seen in the ED at our facility for acute cystitis. At that time, she presented with fever, arthralgia, and myalgia, and urinalysis results pertinent for pyuria, leukocyte esterase, nitrites, and bacteria. She was managed with Tylenol and antibiotics for a presumptive diagnosis of acute cystitis. She was discharged home from ED the same day with a recommendation for primary care provider (PCP) follow-up.

Given her recent ED visit for acute cystitis and her current presentation with fever, hypotension, tachycardia, and pertinent findings on laboratory results and imaging, a preliminary diagnosis of sepsis secondary to pyelonephritis was made. She was started on intravenous fluids hydration and ceftriaxone (IV) 2 g daily for seven days while awaiting culture results.

During her hospital course, it was initially difficult to discern improvement in her symptoms due to inconsistency in reporting, thought to be related to developmental delay. However, it was observed that the patient continued to spike a fever in a quotidian pattern while being on appropriate antibiotics. Her fever was refractory to cooling blankets and Tylenol. We further evaluated the patient’s persistent fever as we began to suspect that it was probably not due to the presumed urinary tract infection (UTI). Blood and urine culture specimens obtained at the presentation continued to show no growth at this time.

A repeat physical examination at the bedside was pertinent for bilateral knee arthralgia that was not observed at the presentation. Examination of her skin did not reveal any rash or lesions. A repeat CBC showed that her WBC was within normal limits despite the fever. A repeat chest X-ray showed no acute interval change from the scan done at the presentation. We then proceeded with a D-dimer, duplex ultrasound of her lower extremity, and a computed tomography angiography (CTA) of the chest to evaluate for venous thromboembolism (VTE). While her D-dimer was markedly elevated at more than 5,036 ng/mL (reference range: <500 ng/mL), her duplex ultrasound showed no evidence of deep vein thrombosis in her bilateral lower extremities and CTA of the chest was negative for pulmonary embolism in her pulmonary vasculature. She underwent transthoracic echocardiography to rule out insidious endocarditis, and it did not show any vegetation.

Given the negative workup for her fever, we considered her pertinent rheumatologic history of SJIA and hypothesized that the patient’s persistent fevers were likely noninfectious but inflammatory in nature. The constellation of her clinical picture, including her quotidian fever, arthralgia, anemia, transaminitis, hypertriglyceridemia, elevated D-dimer, and mild splenomegaly, raised suspicion for MAS, a rare complication of SJIA. We did further ancillary tests, which showed hyperferritinemia and elevated soluble interleukin-2 (IL-2) receptors (Table 4).

**Table 4:** The results of ancillary laboratory tests during the hospital course

Parameters	Result	Reference range
Ferritin	9,563	10 - 291 ng/mL
Soluble interleukin-2 (IL-2)	1919.3	175.3 - 858.2 pg/mL
Antinuclear antibodies	>12	<1
Rheumatoid factor	16	<15

We discussed the case with her outpatient rheumatologist, who had followed the patient since she was diagnosed with SJIA. The rheumatologist reported that the patient had been inconsistent with follow-ups and non-compliant with her weekly methotrexate injections to manage her SJIA. He endorsed that this was most likely a flare with a picture suggestive of MAS and recommended a trial of steroids. The patient was started on IV Solu-Medrol 250 mg daily for two days. Her arthralgia resolved on steroids, and her fever subsided. She made an uneventful recovery and was discharged on oral prednisone 20 mg daily with a referral for outpatient rheumatology follow-up. Her total length of hospital stay was six days.

## Discussion

SJIA (formerly Still’s disease) is a systemic inflammatory disorder characterized by fever, arthritis, rash, and other multivariate extra-articular manifestations. SJIA and AOSD are similar conditions, the only difference is the age at which the disorder is diagnosed; it is called SJIA when the patient is diagnosed before the age of 16 years. MAS is a rare and severe complication of SJIA. The incidence of MAS is estimated at 0.16-0.4/100,000 inhabitants, with a prevalence of 1-24/million inhabitants, and the median age at diagnosis varying from 27 to 36 years [2]. However, historically, MAS has also been described in the literature as a complication of the rheumatic disease of childhood [2,3]. Our patient was a 21-year-old diagnosed with systemic juvenile rheumatoid arthritis; hence, this can be regarded as a classic presentation. MAS is closely associated with HLH, as these two share a similar pathophysiology. Both involve massive macrophage activation with a resulting cytokine storm that can potentially cause multi-organ failure if untreated [3,4].

While HLH is a more general term with a myriad of triggers like malignancies, viral infections, or autoimmune disease, MAS is specifically used when referring to rheumatology-related HLH. However, in some studies, HLH and MAS have been used interchangeably [5-8]. Amongst the related autoimmune/rheumatology disorders, MAS is most frequently associated with AOSD [3,5]. In our report, we intentionally used HLH when describing classifications and diagnostic criteria. Although both disorders are similar, the extant literature primarily focuses on HLH as the parent disorder. The pathophysiology of HLH/MAS has been described as excessive macrophage and T-cell activation with resulting uncontrolled release of interleukin (IL)-1B, IL-6, IL-18, and interferon-gamma. HLH is subdivided into primary or familial HLH and secondary or reactive HLH [6-8]. In primary HLH, the uncontrolled proliferation of T cells and macrophages has been linked to decreased natural killer (NK) cell and cytotoxic T cell function, often due to mutations in the gene encoding perforin, a protein that induces apoptosis of target cells. There are also variations seen with mutations in the MUNC13-4 gene, which encodes a protein that releases perforin, thus reducing their ability to kill target cells [6,7]. Patients in secondary HLH tend to have less severe clinical presentations than in primary HLH, which can occur at any age. However, mortality in this group is still considered high [7].

Besides the excessive immune activation, the immunopathophysiology of MAS also involves the failure of NK cells and cytotoxic T lymphocytes (CTLs) to kill these rogue cells and thus halt the cytokine storm [6]. It is pertinent to mention that AOSD/SJIA has a pathophysiological process similar to HLH. There are similarities in the symptomatology, such as fever, enlargement of the liver and spleen, and elevated serum ferritin levels, all of which appear to present as confounding elements [3]. The Yamaguchi criteria used in diagnosing AOSD requires the presence of five features, with at least two being major diagnostic criteria. Major criteria include fever ≥ 39ºC for ≥ one week, arthralgia/arthritis for ≥ two weeks, salmon-colored rash, and WBC (≥10K + ≥80% polymorphonuclear neutrophils), and the minor features include sore throat, lymphadenopathy, hepatosplenomegaly, aspartate aminotransferase/alanine aminotransferase, lactate dehydrogenase, and negative antinuclear antibody and rheumatoid factor. Although not part of the diagnostic criteria of AOSD, hyperferritinemia is also commonly seen in AOSD, although quantitatively less compared to the MAS spectrum [7].

The current diagnostic criteria for HLH employs the use of five out of nine of the following findings: fever ≥ 38.5°C, splenomegaly, cytopenias in ≥ two blood lineages, hypertriglyceridemia and/or hypofibrinogenemia, hemophagocytosis in bone marrow, spleen, lymph node, or liver, ferritin > 500 ng/mL (although >3000 ng/mL is more indicative of HLH), low or absent NK cell activity, elevated sCD25, and elevated CXCL9. As can already be inferred, given the overlapping symptomatology, our index patient had symptoms equally suspicious for an AOSD/SJIA flare, but given her markedly elevated ferritin (9,563 ng/mL) and the overall clinical picture, MAS was more likely. She also had a constellation of symptoms that were appropriately worrisome for sepsis, given that she presented with fever, chills, hypotension, tachypnea, marked leukocytosis, recent UTI, and CT imaging documenting pyelonephritis as the likely source of infection. However, given the unremarkable urinalysis findings at presentation, absence of lower urinary tract symptoms, negative blood and urine cultures, lack of improvement with antibiotics, and improvement with steroids, it is likely that the UTI, if present, was not the primary driving factor. But even more interesting and worthy of consideration is that MAS/HLH can be triggered by infections, so a preceding UTI in our index patient may have triggered a MAS picture. A similar study was conducted by Barman et al. on a 21-year-old female with HLH secondary due to AOSD. However, in that report, the patient had no prior pertinent rheumatological history and was newly diagnosed with AOSD complicated by HLH at presentation [9,10].

Given the nonspecific nature of the syndrome, some clinicians believe that a MAS/HLH diagnosis should not be categorically made without evidence of hemophagocytosis seen on a biopsy sample. However, given the life-threatening nature of MAS, and the fact that hemophagocytosis is not always noted on biopsy, we recommend keeping a high index of suspicion for MAS and commencing treatment even before biopsy results [11].

Prompt diagnosis and early treatment are recommended, with addressing the triggering factor if identified, administering high-dose steroids, and, in refractory cases, giving biological agents [11]. The treatment usually involves intravenous methylprednisolone pulse therapy or cyclosporine if a response to steroids is not evident within 24-48 hours [5,9]. Induction therapy consists of etoposide (twice weekly during the first two weeks and then weekly) in combination with dexamethasone. Central nervous system disease, however, is treated with intrathecal methotrexate [12]. However, caution is exercised while using etoposide due to the risk of severe myelosuppression and hepatic and renal side effects [5]. Antithymocyte globulin (ATG) can also be used, and in Epstein-Barr virus (EBV), one might consider rituximab in combination with traditional HLH therapies [5,13,14]. Anakinra is a recombinant form of human interleukin-1 receptor antagonist (IL-1Ra), and it blocks the biological activity of both IL-1α and IL-1β by competitively inhibiting their binding to the interleukin-1 receptor (IL-1R) [6]. Biologic agents such as anakinra or emapalumab directly neutralize cytokines [14]. Anakinra is usually effective in corticosteroid dependence (or when cyclosporine and etoposide fail to control the disease), and tocilizumab is preferred in cases of inflammatory rheumatism [1,15].

Recently, a web-based online calculator has been developed called the HLH probability calculator (HScore), and this might prove to be a helpful diagnostic tool [16]. It was developed in adult patients and consists of graded clinical and laboratory parameters. To prevent overtreatment and unnecessary toxicity in adults, individualized tailoring of treatment duration, dose reductions, and an age-dependent modified diagnostic approach is to be considered [17].

## Conclusions

MAS is challenging to diagnose and manage due to its overlap in presentation with many other clinical conditions, which presents the conundrum of a very broad differential diagnosis. With this case report, we set out to highlight several key teaching points in the diagnosis and management of MAS. The first is the recognition that MAS diagnosis can be made entirely on a clinical basis, which means that in the absence of a biopsy result (or identification of HLH gene mutation in genetic testing), the diagnosis hinges on certain nonspecific clinical and laboratory parameters. Broadly speaking, while the extant diagnostic criteria likely allow for increased sensitivity in identifying MAS in patients who will benefit from life-saving therapy, an inadvertently low diagnostic specificity raises genuine concerns for an increase in false-positive rates. What we need is a test or diagnostic protocol that is highly sensitive, but also highly specific. This broad differential diagnostic quagmire becomes crucial in deciding on who and when to initiate treatment. The cornerstone of MAS therapy involves suppression of the ongoing life-threatening inflammation with medications that have worrisome adverse effects profiles. We believe that to improve the outcome associated with this disease, there needs to be a better understanding of the disease pathogenesis, a high index of suspicion to make a prompt diagnosis, the development of more predictive diagnostic tests, the development of standardized treatment protocols, and newer treatment modalities with fewer toxicities.
